# The Relationship Between Smoking and Multiple Sclerosis Severity in Saudi Arabia

**DOI:** 10.7759/cureus.24181

**Published:** 2022-04-16

**Authors:** Seraj Makkawi, Fahad A AlHarbi, Nedaa Alsulaimani, Reem Brashi, Renad Melebary, Shuaa Aljabri, Khalid F Altowairqi, Albaraa F Ashoor, Amal Alkhotani

**Affiliations:** 1 College of Medicine, King Saud Bin Abdulaziz University for Health Sciences, Jeddah, SAU; 2 Research and Development, King Abdullah International Medical Research Center, Jeddah, SAU; 3 Department of Medicine, Ministry of National Guard Health Affairs, Jeddah, SAU; 4 Department of Medicine, Faculty of Medicine, Umm Al-Qura University, Makkah, SAU

**Keywords:** progression, relapse, saudi arabia, smoking, multiple sclerosis

## Abstract

Introduction

Multiple sclerosis (MS) is an autoimmune disease that can be disabling to patients. Smoking has been proposed to be a risk factor for MS and to increase the risk of progression of the disease and its severity. However, it is still not clear how smoking affects people with MS (PwMS) regarding disease phenotype, symptoms, relapses, course, and disability. The aim of this study is to investigate the effect of smoking on PwMS in Saudi Arabia.

Methods

This is an online questionnaire-based cross-sectional study. PwMS were randomly contacted through different MS societies and associations to participate in the study. The questionnaire inquired about demographics, MS phenotype and severity, and smoking status of the participants. Data were collected between May 30, 2021, and July 5, 2021.

Results

Four hundred twenty-nine PwMS participated in the study. The mean age was 33.7, with a mean disease duration of 8.1 years. About 61.1% of the participants were female. About 62.2% did not know the specific MS phenotype they have. About 35.7% were current or previous smokers, with a mean smoking duration of 13.9 years. Smoking was significantly associated with the presence of multiple MS symptoms (p-value = 0.009) and their number (p-value = 0.050). In addition, there was a significant positive correlation between pack-years smoking and the number of MS symptoms with a Pearson's r value of 0.165 (p-value = 0.001). No significant associations were found between smoking and recent relapses and disease progression, disability in terms of walking, needing a cane, or needing a wheelchair.

Conclusion

Smoking was shown to have a significant effect on the number of symptoms experienced by PwMS. Higher pack-years of smoking correlates positively and significantly with a higher number of MS symptoms. Further studies to examine these relations are hence warranted.

## Introduction

Multiple sclerosis (MS) is the most common autoimmune disease affecting the central nervous system. Its worldwide prevalence has been reported to increase from 2.3 million in 2013 to 2.8 million (36/100,000) in 2020 [1]. The disease is categorized into three main phenotypes based on the clinical course and symptomatology: relapsing-remitting multiple sclerosis (RRMS), primary progressive multiple sclerosis (PPMS), and secondary progressive multiple sclerosis (SPMS). RRMS is the most common phenotype, approximately affecting 85% of patients [2]. MS is a major cause of non-traumatic neurological disability [3]. Several lifestyle and nutritional interventions are proposed to decrease the risk of disability associated with MS and the progression of the disease, such as vitamin D supplementation, exercise, plant-based diet, and smoking cessation [4]. A lack of control over these factors in addition to other factors is thought to be responsible for an increased prevalence of MS in the Arabian Gulf region [5]. MS is also shown to be more severe in Saudi Arabia than in other countries [6], with an overall MS prevalence of 40.4/100,000 in the country [7].

Smoking is considered one of the main risk factors for multiple sclerosis [8,9]. A systematic review showed strong evidence of the causal role of smoking in MS, using Hill’s criteria [10]. Smoking was also associated with higher levels of MS physical pain and worse long-term disease prognosis [11]. Smoking is also hypothesized to play a role in MS progression [10,12]. In addition, smoking is shown to increase the frequency of relapses, trigger the onset of MS symptoms, and alter the activity of MS disease-modifying drugs. All of which could lead to the negative role of smoking on MS [13]. The relationship between smoking and MS severity, course, and progression in Saudi Arabia has not been investigated yet. This study aims to provide an insight into this relationship and investigate the role of smoking on the increased severity and prevalence of MS in the country.

## Materials and methods

Design and setting

This is an online questionnaire-based cross-sectional study done in Saudi Arabia. Simple random sampling technique was implemented. MS societies and associations were contacted to randomly send a link of the questionnaire to registered people with MS (PwMS) study. This study has been approved by Umm Al-Qura University (UQU) institutional review board (IRB) No. HAPO-02-K-012-2021-03-625.

The link displayed an explanation of the purpose of the study and its targeted population, IRB approval, an informed consent form ensuring the anonymity of participants, and a choice to either agree or disagree to participate in the study. Data were collected between May 30, 2021, and July 5, 2021. The targeted sample size was calculated to be 370 using “sample size for estimating a proportion” equation described by Thompson [14]. A 95% confidence interval was implanted for the calculation, with an error proportion of 5%, population proportion of 50%, and an estimated total population of 10,000.

Questionnaire and variables

We utilized Google Forms (Google, Mountain View, California, United States) to design and distribute our questionnaire. It consisted of three sections. The first section included different demographics of the participants. The second section included questions asking about MS severity: disease phenotype, year of diagnosis, relapses in the last year and any new MRI findings, the ability to walk for different distances in a flat terrain, any walking aids or wheelchair use, and inquired about current symptoms (e.g., dysarthria, weakness, ataxia). Self-reported disability status scale (SRDSS) was used to estimate the Expanded Disability Status Scale (EDSS) score categories (≤3.5, 4-6.5, ≥7) [15]. The third section asked about smoking status (smokers, previous smokers, or non-smokers). It also inquired about duration of smoking (few months, less than five years, five to 10 years, more than 10 years), year of smoking cessation for previous smokers, age when smoking started, how many cigarettes a participant smokes daily, and about the regularity of smoking. Pack-years of smoking was calculated for each participant, and heaviness of smoking was classified as light smokers (>0-20 pack-years), moderate smokers (>20-40 pack-years), and heavy smokers (>40 pack-years). The questionnaire was distributed in Arabic, and the results were later translated to English for analysis and publication purposes.

Data analysis

Data were analyzed using Statistical Package for the Social Sciences (SPSS) software version 20.0 (IBM, Chicago, Illinois, United States). Descriptive analyses were used to present participants' demographics, disease phenotype, and smoking status. The association between categorical variables was examined using chi square test. Correlation between continuous variables was examined using Pearson’s r. A 95% confidence interval and a significance level of 5% were utilized.

## Results

Demographics

A total of 429 participants completed the questionnaire. Two hundred sixty-two (61.1%) of the sample were females. The mean age was 33.7 ± 9.6 years. Non-smoker participants were 276 (64.3%) of the total sample, while current smokers were 99 (23.1%), and previous smokers were 54 (12.6%). The mean duration of smoking was 13.9 ± 8.9 years, and the mean pack-years of smoking was 13.0 ± 15.3 (Table 1). 

**Table 1:** Demographics and descriptive statistics Light smoker: >0-20 pack-years, moderate smoker: >20-40 pack-years, and heavy smoker: >40 pack-years. SD: standard deviation, SR: Saudi riyal.

Continuous variables	Mean	SD
Age	33.7	9.6
Age at start of smoking	19.3	5.0
Duration of smoking	13.9	8.9
Number of cigarettes/days	16.1	13.6
Pack-years of smoking	13.0	15.3
Categorical variables	n	%
Gender		
Male	167	38.9
Female	262	61.1
Nationality		
Saudi	387	90.2
Non-Saudi	89	39.6
Education		
Less than high school	15	3.5
High school	115	26.8
Bachelor	260	60.6
Master	36	8.4
PhD or higher	3	0.7
Income		
Less than 5,000 SR	221	51.5
5,000-10,000 SR	114	26.6
10,000-15,000 SR	56	13.1
More than 15,000 SR	38	8.9
Smoking status		
Non-smoker	275	64.1
Ever smoker	153	35.7
Previous smoker	54	12.6
Current smoker	99	23.1
Smoking heaviness		
Non-smoker	275	64.1
Light smoker	82	19.1
Moderate smoker	17	4.0
Heavy smoker	6	1.4
Unknown	49	11.4
Total	429	100.0

MS phenotypes were reported as follows: relapsing-remitting MS (RRMS) in 106 participants (24.7%), primary progressive MS (PPMS) in 33 (7.7%), and secondary progressive MS (SPMS) in 23 (5.4%). However, 267 (62.2%) participants did not know what type of MS they have. The mean duration of the disease was 8.1 ± 6.7 years. The number of participants who had a relapse in the last year was 157 (36.6%), and 152 (35.4%) had a new MRI lesion. Regarding the progression of the disease, 138 participants (32.2%) had a progression in the last year. One hundred thirteen (26.3%) were unable to walk for 10 m on flat ground. Ninety (21.0%) participants reported they need a cane to walk, and 50 (11.7%) reported the need of a wheelchair. One hundred sixty-eight (39.2%) participants had an SRDSS score of 3.5 or less, 148 (34.5%) had a score between 4 and 6.5, and 113 (26.3%) had a score of 7 or more. Finally, 361 (84.1%) had at least one MS symptom at the time of participation in the study, and 111 (25.9%) had more than three symptoms (Table 2).

**Table 2:** Multiple sclerosis outcomes Light smoker: >0-20 pack-years, moderate smoker: >20-40 pack-years, and heavy smoker: >40 pack-years. SD: standard deviation, MS: multiple sclerosis, RRMS: relapsing-remitting MS, PPMS: primary progressive MS, SPMS: secondary progressive MS, SRDSS: self-reported disability status scale.

Continuous variables	Mean	SD
Disease duration	8.1	6.7
Relapses in last year	1.0	2.0
Number of current symptoms	2.3	1.6
Categorical variables	n	%
MS phenotype		
RRMS	106	24.7
PPMS	33	7.7
SPMS	23	5.4
Don't know	267	62.2
Relapse in the last year		
Yes	157	36.6
No	210	49.0
Don't know	62	14.5
New MRI lesion in the last year		
Yes	152	35.4
No	149	34.7
Don't know	128	29.8
Progression in the last year		
Yes	138	32.2
No	235	54.8
Don't know	56	13.1
Ability to walk on a flat terrain		
More than 500 m	175	40.8
10-500 m	141	32.9
Less than 10 m	113	26.3
Need a cane to walk		
Yes	90	21.0
No	339	79.0
Need a wheelchair to mobilize		
Yes	50	11.7
No	379	88.2
SRDSS		
3.5 or less	168	39.2
4-6.5	148	34.5
7 or more	113	26.3
Presence of at least one current symptom		
Yes	361	84.1
No	68	15.9
Presence of multiple current symptoms		
Three or less symptoms	318	74.1
More than three symptoms	111	25.9
Total	429	100.0

Relation between smoking and MS

Phenotype

The statistical analysis showed a significant difference between ever-smokers (current smokers and previous smokers) and non-smokers in terms of their MS phenotype (p-value = 0.003) (Table 3). This difference persisted when tested between smokers, previous smokers, and non-smokers (p-value = 0.008). In addition, there was a significant difference between light smokers, moderate smokers, and heavy smokers in terms of disease phenotype (p-value = 0.01) (Table 4). However, there was no significant difference between different smoking durations and disease phenotype (p-value = 0.31) (Table 4). 

**Table 3:** Smoking status association with other variables p-values < 0.05 are given in bold. RRMS: relapsing-remitting multiple sclerosis, PPMS: primary progressive multiple sclerosis, SPMS: secondary progressive multiple sclerosis, SRDSS: self-reported disability status scale.

	Non-smoker		Ever-smoker		Total		p-value
	n	%	n	%	n	%	
Phenotype							
RRMS	56	20.4%	50	32.7%	106	24.8%	0.003
PPMS	16	5.8%	16	10.5%	32	7.5%	
SPMS	14	5.1%	9	5.9%	23	5.4%	
Don't know	189	68.7%	78	51.0%	267	62.4%	
Relapses in last year							
Yes	108	39.3%	49	32.0%	157	36.7%	0.23
No	126	45.8%	83	54.2%	209	48.8%	
Don't know	41	14.9%	21	13.7%	62	14.5%	
New MRI lesions in last year							
Yes	95	34.5%	56	36.6%	151	35.3%	0.66
No	100	36.4%	49	32.0%	149	34.8%	
Don't know	80	29.1%	48	31.4%	128	23.9%	
Progression in last year							
Yes	89	32.4%	49	32.0%	138	32.2%	0.09
No	157	57.1%	77	50.3%	234	54.7%	
Don't know	29	10.5%	27	17.6%	56	13.1%	
Ability to walk different distances on a flat terrain							
More than 500 m	104	37.8%	71	46.4%	175	40.9%	0.18
10-500 m	93	33.8%	48	31.4%	141	32.9%	
Less than 10 m	78	28.4%	34	22.2%	112	26.2%	
Need a cane to walk							
Yes	53	19.3%	37	24.2%	90	21.0%	0.23
No	222	80.7%	116	75.8%	338	79.0%	
Need a wheelchair to mobilize							
Yes	34	12.4%	16	10.5%	50	11.7%	0.55
No	241	87.6%	137	89.5%	378	88.3%	
SRDSS							
3.5 or less	100	36.4%	68	44.4%	168	39.3%	0.20
4-6.5	97	35.3%	51	33.3%	148	34.6%	
7 or more	78	28.4%	34	22.2%	112	26.2%	
Presence of at least one current symptoms							
Yes	232	84.4%	129	84.3%	361	84.3%	0.98
No	43	15.6%	24	15.7%	67	15.7%	
Presence of multiple current symptoms							
Three or less symptoms	215	78.2%	102	66.7%	317	74.1%	0.009
More than three symptoms	60	21.8%	51	33.3%	111	25.9%	

**Table 4:** Smoking duration association with other variables p-values < 0.05 are given in bold. RRMS: relapsing-remitting multiple sclerosis, PPMS: primary progressive multiple sclerosis, SPMS: secondary progressive multiple sclerosis, SRDSS: self-reported disability status scale.

	Non-smoker		Few months		Less than five years		Five to 10 years		More than 10 years		Total		p-value
	n	%	n	%	n	%	n	%	n	%	n	%	
Phenotype													
RRMS	56	20.4%	3	23.1%	9	45.0%	6	24.0%	31	34.4%	105	24.8%	0.06
PPMS	16	5.8%	1	7.7%	2	10.0%	4	16.0%	9	10.0%	32	7.6%	
SPMS	14	5.1%	1	7.7%	0	0.0%	2	8.0%	4	4.4%	21	5.0%	
Don't know	189	68.7%	8	61.5%	9	45.0%	13	52.0%	46	51.1%	265	62.6%	
Relapses in last year													
Yes	108	39.3%	5	38.5%	7	35.0%	10	40.0%	25	27.8%	155	36.6%	0.74
No	126	45.8%	6	46.2%	11	55.0%	12	48.0%	52	57.8%	207	48.9%	
Don't know	41	14.9%	2	15.4%	2	10.0%	3	12.0%	13	14.4%	61	14.4%	
New MRI lesions in last year													
Yes	95	34.5%	3	23.1%	12	60.0%	9	36.0%	31	34.4%	150	35.5%	0.11
No	100	36.4%	6	46.2%	5	25.0%	12	48.0%	25	27.8%	148	35.0%	
Don't know	80	29.1%	4	30.8%	3	15.0%	4	16.0%	34	37.8%	125	29.6%	
Progression in last year													
Yes	89	32.4%	2	15.4%	9	45.0%	7	28.0%	28	31.1%	135	31.9%	0.056
No	157	57.1%	9	69.2%	6	30.0%	14	68.0%	44	48.9%	233	55.1%	
Don't know	29	10.5%	2	15.4%	5	25.0%	1	4.0%	18	20.0%	55	13.0%	
Ability to walk different distances on a flat terrain													
More than 500 m	104	37.8%	4	30.8%	8	40.0%	15	60.0%	41	45.6%	172	40.7%	0.26
10-500 m	93	33.8%	7	53.8%	5	25.0%	7	28.0%	28	31.1%	140	33.1%	
Less than 10 m	78	28.4%	2	15.4%	7	35.0%	3	12.0%	21	23.3%	111	26.2%	
Need a cane to walk													
Yes	53	19.3%	1	7.7%	7	35.0%	2	8.0%	26	28.9%	89	21.0%	0.038
No	222	80.7%	12	92.3%	13	65.0%	23	92.0%	64	71.1%	334	73.0%	
Need a wheelchair to mobilize													
Yes	34	12.4%	1	7.7%	4	20.0%	1	4.0%	9	10.0%	49	11.6%	0.49
No	241	87.6%	12	92.3%	16	80.0%	24	96.0%	81	90.0%	374	88.4%	
SRDSS													
3.5 or less	100	36.4%	4	30.8%	8	40.0%	15	60.0%	38	42.2%	165	39.0%	0.27
4-6.5	97	35.3%	7	53.8%	5	25.0%	7	28.0%	31	34.4%	147	34.8%	
7 or more	78	28.4%	2	15.4%	7	35.0%	3	12.0%	21	23.3%	111	26.2%	
Presence of at least one current symptoms													
Yes	232	84.4%	13	100.0%	18	90.0%	23	92.0%	73	81.1%	359	84.9%	0.31
No	43	15.6%	0	0.0%	2	10.0%	2	8.0%	17	18.9%	64	15.1%	
Presence of multiple current symptoms													
Three or less symptoms	215	78.2%	6	46.2%	10	50.0%	22	88.0%	61	67.8%	314	74.2%	0.001
More than three symptoms	60	21.8%	7	53.8%	10	50.0%	3	12.0%	29	32.2%	109	25.8%	

Presence of MS Symptoms

There was no significant association between smoking status and the current presence of symptoms (p-value = 0.98) (Table 3). Similarly, there was no significant association between smoking duration and the current presence of symptoms (p-value = 0.31) (Table 4). Nor was there a significant association between heaviness of smoking and the current presence of symptoms (p-value = 0.34) (Table 5).

**Table 5:** Smoking heaviness association with other variables p-values < 0.05 are given in bold. Light smoker: >0-20 pack-years, moderate smoker: >20-40 pack-years, and heavy smoker: >40 pack-years. RRMS: relapsing-remitting multiple sclerosis, PPMS: primary progressive multiple sclerosis, SPMS: secondary progressive multiple sclerosis, SRDSS: self-reported disability status scale.

	Non-smoker		Light smoker		Moderate smoker		Heavy smoker		Total		p-value
	n	%	n	%	n	%	n	%	n	%	
Phenotype											
RRMS	58	20.9%	28	34.1%	7	41.2%	1	16.7%	94	24.6%	0.010
PPMS	16	5.8%	12	14.6%	2	11.8%	0	0.0%	30	7.9%	
SPMS	14	5.1%	5	6.1%	0	0.0%	0	0.0%	19	5.0%	
Don't know	189	68.2%	37	45.1%	8	47.1%	5	83.3%	239	62.6%	
Relapses in last year											
Yes	108	39.0%	25	30.5%	4	23.5%	1	16.7%	138	36.1%	0.23
No	128	46.2%	48	58.5%	10	58.8%	5	83.3%	191	50.0%	
Don't know	41	14.8%	9	11.0%	3	17.6%	0	0.0%	53	13.9%	
New MRI lesions in last year											
Yes	95	34.3%	31	37.8%	6	35.3%	1	16.7%	133	34.8%	0.08
No	102	36.8%	31	37.8%	2	11.8%	1	16.7%	136	35.6%	
Don't know	80	28.9%	20	24.4%	9	52.9%	4	66.7%	113	29.6%	
Progression in last year											
Yes	89	32.1%	23	28.0%	7	41.2%	1	16.7%	120	31.4%	0.19
No	159	57.4%	45	54.9%	6	35.3%	3	50.0%	213	55.8%	
Don't know	29	10.5%	14	17.1%	4	23.5%	2	33.3%	49	12.8%	
Ability to walk different distances on a flat terrain											
More than 500 m	106	38.3%	42	51.2%	5	29.4%	4	66.7%	157	41.1%	0.10
10-500 m	93	33.6%	25	30.5%	4	23.5%	1	16.7%	123	32.2%	
Less than 10 m	78	28.2%	15	18.3%	8	47.1%	1	16.7%	112	26.7%	
Need a cane to walk											
Yes	53	19.7%	22	26.8%	7	41.2%	2	33.3%	84	22.0%	0.08
No	224	80.9%	60	73.2%	10	58.8%	4	66.7%	298	78.0%	
Need a wheelchair to mobilize											
Yes	34	12.3%	9	11.0%	3	17.6%	1	16.7%	47	12.3%	0.87
No	243	87.7%	73	89.0%	14	82.4%	5	83.3%	335	87.7%	
SRDSS											
3.5 or less	102	36.8%	40	48.8%	5	29.4%	3	50.0%	150	39.3%	0.18
4-6.5	97	35.0%	27	32.9%	4	23.5%	2	33.3%	130	34.0%	
7 or more	78	28.2%	15	18.3%	8	47.1%	1	16.7%	102	26.7%	
Presence of at least one current symptoms											
Yes	234	84.5%	68	82.9%	17	100.0%	5	83.3%	324	84.8%	0.34
No	43	15.5%	14	17.1%	0	0.0%	1	16.7%	58	15.2%	
Presence of multiple current symptoms											
Three or less symptoms	216	78.0%	56	68.3%	10	58.8%	2	33.3%	284	74.3%	0.012
More than three symptoms	61	22.0%	26	31.7%	7	41.2%	4	66.7%	98	25.7%	

However, there was a significant difference between ever-smokers and non-smokers in terms of the presence of multiple MS symptoms (p-value = 0.009) (Table 3). This difference persisted when tested between smokers, previous smokers, and non-smokers (p-value = 0.03). Moreover, the odds ratio of having multiple symptoms for ever-smokers compared to non-smokers was 1.79 (95% CI: 1.15-2.78). Similarly, there was a significant difference between different smoking durations in terms of the presence of multiple MS symptoms (p-value = 0.001) (Table 4) and between light smokers, moderate smokers, and heavy smokers in the same terms (p-value = 0.01) (Table 5).

There was a significant positive correlation between the number of pack-years smoked and the number of current MS symptoms (r = 0.165, p-value = 0.001). Smoking duration was also significantly correlated with number of current symptoms (r = 0.107, p-value = 0.02). However, the duration since smoking cessation did not correlate significantly with the number of symptoms (r = 0.152, p-value = 0.31). Finally, the number of cigarettes smoked per day correlated significantly with the number of current MS symptoms (r = 0.128, p-value = 0.01).

Relapses and Progression

There was no significant association between smoking status and relapse in the last year (p-value = 0.23). Neither was there an association with new MRI lesions (p-value = 0.66) nor progression in the last year (p-value = 0.09) (Table 3). Similarly, there was no significant association between smoking duration and relapses in the last year (p-value = 0.74), new MRI lesion (p-value = 0.11), and progression in the last year (p-value = 0.056) (Table 4). Also, there was no significant association between heaviness of smoking in terms of relapses in the last year (p-value = 0.23), new MRI lesion (p-value = 0.08), and progression in the last year (p-value = 0.19) (Table 5). Similarly, correlation analysis showed no significant correlation between smoking duration and relapses in the past year (r = 0.03, p-value = 0.47), number of cigarettes smoked per day and relapses (r = 0.02, p-value = 0.59), or pack-years smoked and relapses (r = -0.03, p-value = 0.50).

Walking

There was no significant association between smoking status and ability to walk on flat ground for different distances (p-value = 0.18) (Table 3). Similarly, there was no significant association between smoking duration and ability to walk different distances (p-value = 0.26) (Table 4). There was no significant association between heaviness of smoking and ability to walk different distances either (p-value = 0.10) (Table 5).

Cane

There was no significant association between smoking status and the need of a cane (p-value = 0.23) (Table 3). In contrast, there was a significant difference between different smoking durations in terms of the need to use a cane (p-value = 0.03) (Table 4). However, there was no significant association between heaviness of smoking and the need to use a cane (p-value = 0.08) (Table 5).

Wheelchair

There was no significant association between smoking status and the need of a wheelchair (p-value = 0.55) (Table 3). Similarly, there was no significant association between smoking duration and the need of a wheelchair (p-value = 0.49) (Table 4). Also, there was no significant association between heaviness of smoking and the need to use a wheelchair (p-value = 0.87) (Table 4).

Self-Reported Disability Status Scale

There was no significant association between smoking status and SREDSS (p-value = 0.20) (Table 3). Similarly, there was no significant association between smoking duration and SREDSS (p-value = 0.27) (Table 5). Also, there was no significant association between heaviness of smoking and SREDSS (p-value = 0.18) (Table 5).

## Discussion

Our study showed that most PwMS do not know their disease phenotype, which is similar to what another study done in Jeddah reported [16]. This could reflect a lack of patient awareness of their disease or a lack of effective communication and education by physicians. In a study investigating patients’ awareness of MS in Saudi Arabia, it was reported that only 37% knew the correct definition of RRMS. Our study also showed that the proportion of PwMS who currently smoke (23.1%) is higher than the proportion of current smokers in the general Saudi population (19.8%) [17]. A study investigating awareness of smoking risk among PwMS in Jeddah also showed a high prevalence of active smokers (34.6%). It was also shown that most patients did not know whether smoking is a risk factor for MS or disagreed to this [16]. All of this shows that, indeed, smoking is prevalent among PwMS and can be a strong risk factor for the disease in Saudi Arabia. A previous case-control study investigating MS risk factors in Saudi Arabia also found smoking to be a strong risk factor [18].

In this study, our aim was to investigate the specific effects smoking has on MS severity and progression. We found that smoking is significantly associated with the presence of multiple MS symptoms. This association was evident in all statistical tests we performed. In addition, there was a significant difference between smoking duration and the need to use a cane to walk. This is consistent with what was reported in a previous review, which showed smoking to be associated with higher rates of disease activity, greater disability burden, more relapses, and faster rates of brain atrophy [19,20].

Although smoking status was significantly associated with MS phenotype, the huge proportion of people not knowing their specific phenotype prompts a cautious interpretation of this association. It is important, however, that this association does not go unnoticed, as it could indicate that smoking has a role in the type of MS at onset or the later progression of RRMS to SPMS. This was found in a study done on over 1,400 PwMS, which showed smokers to be more likely to have PPMS and to progress from RRMS to SPMS [21].

Other variables, such as relapses in the last year, new MRI lesions, and SRDSS, were not associated with smoking on any statistical test we performed. The limited effects of smoking only on the presence of multiple symptoms, but not on disease progression or the number of MRI lesions, can be attributed to the high percentages of patients who do not know their clinical status accurately. It was shown in another study that only 42.6% of patients reported that they were well informed about their disease [22]. 

Multiple MS symptoms could significantly decrease patients’ quality of life. They can lead to more fatigue, more depressed mood, and more physical disability. These three clinical domains were constantly correlated to low health-related quality of life [23-25]. This study had several limitations. The cross-sectional design could lead to recall bias, and the online nature of the study could not allow the surveyor to help with any difficulty the participants face when answering the questionnaire. These effects could be responsible for many patients answering “don’t know” to several questions, which could have resulted in decreased power of the study.

## Conclusions

Smoking status, heaviness, and duration were all significantly associated with the presence of multiple MS symptoms. The number of pack-years smoked was significantly correlated with the higher number of current symptoms. Smoking duration was also significantly associated with the need to use a cane to walk. Although smoking was not shown to be associated with other variables (such as relapses, new MRI lesions, and SRDSS), further studies are needed to confirm the absence of association. Also, more studies are needed to further investigate the specific symptoms associated with smoking.
